# Midpalatal suture: evaluation of the morphological maturation stages via bone density

**DOI:** 10.1186/s40510-018-0232-2

**Published:** 2018-08-13

**Authors:** Dani Abo Samra, Rania Hadad

**Affiliations:** Department of Orthodontics and Dentofacial Orthopaedics, Faculty of Dental Medicine, Damascus University, AlMazzah Street, Damascus, Syria

**Keywords:** Midpalatal suture, Morphological maturation stages, Bone density

## Abstract

**Background:**

To investigate the relationship between the morphological maturation stages of the midpalatal suture and its bone densities.

**Methods:**

The sample consisted of 91 subjects aged 8–18 years who underwent cone beam computed tomography. All images were examined to classify morphological maturation of the midpalatal suture to five groups according to Angelieri et al. Bone density of the midpalatal suture was measured at the maxillary and palatal regions. Kruskal-Wallis and Mann-Whitney *U* tests were used to analyze the difference between groups.

**Results:**

Bone density of the midpalatal suture was significantly higher in the palatal region in E stage and in the maxillary region in D and E stages.

**Conclusions:**

It is concluded that the change in bone density of the midpalatal suture between the morphological maturation stages supports their reliability in clinical application.

## Background

Rapid maxillary expansion (RME) is a routine procedure in orthodontic treatment for correction of constricted maxillary in growing patients [1]. After adolescence, it is usually necessary to perform surgically assisted RME (SARME) to reduce the resistance to expansion [1, 2]. The time point to shift from RME to SARME is not clear enough especially in young adults [3–5]. Most studies suggest that RME should be presented before puberty [6, 7]. In contrast, other case reports have shown a successful expansion by RME without surgical weakness in adult patients [8–10].

Midpalatal suture (MPS) is one of the most important regions of resistance to the expansion of the maxillary [11, 12]. Other contributory resistance regions to the maxillary expansion were demonstrated in the literature, such as the circummaxillary sutures (zygomaticomaxillary, zygomaticotemporal, and pterygopalatine sutures) [13, 14] and maxillary buttresses (piriform aperture, zygomatic buttresses, and pterygoid junctions) [3, 15].

Since chronological age is unreliable for determining the developmental status of the suture during growth [16–19], understanding individual variability in the developmental status of the MPS is essential in identifying prospectively which late adolescent or young adult patient can have RME as a less-invasive alternative to SARME [4, 20].

Those individual variability include obliteration index (OI), MPS morphology (MPSM), and MPS density (MPSD) [3, 4, 17, 18, 20–22].

Although initial studies of the MPS concerned about OI as the limiting factor for the treatment choice [23], none of them found a direct relationship between OI and chronologic or skeletal age. Furthermore, OI remains low even in older groups. Therefore, OI was not recognized as a valid reason for the increased transversal expansion resistance in adult patients [17–19].

A new classification method of MPS morphology presented by Angelieri et al. [4] divided the maturation stages dichotomously into A–C and D or E, which might help to avoid the side effects of RME failure or unnecessary SARME. They suggested that this description represents the margin between the viability and non-viability of conservative RME [4], and they concluded that an individual assessment of the midpalatal suture in adults with cone beam computed tomography (CBCT) should be undertaken to avoid an unnecessary surgery for patients [22], for instance, if stage C is observed, a conventional RME procedure would have a good prognosis even in patients over 15 years [24].

On the other hand, because of the proportional relation between the bone density and its resistance to fractures [25], and the increased MPSD with age [18, 26], it has been suggested that MPSD is the most reliable explanation of the increase in maxillary resistance to expansion with age. Similarly, Grünheid et al. [5] concluded that MPDS has the potential to be a useful clinical predictor of the skeletal response to RME. Acar et al. [3] found a highly significant correlation between MPS and the intermolar angle increase (which may reflect alveolar bending and decrease of skeletal effects of RME).

The purpose of this study is aimed at evaluating the relationship between the morphological maturation stages and MPDS to support the reliability of these stages as a determining factor in indicating the appropriate treatment protocol.

## Methods

This study was approved by the ethics committee at the Ministry of Higher Education in Syria (protocol **#**2128).

The sample size was calculated using the G*power 3.1.7 program according to the following assumptions: effect size of 0.49 (depending on the result of a pilot study included 25 patients), the statistical test to be used is one-way ANOVA with a statistical power of 95% and a significance level of 0.05, and a sample size of at least 85 patients was necessary. All available CBCT scans of patients in the archive matching the inclusion criteria were included in the study, and the sample size was 91 CBCT scans.

CBCT images were obtained retrospectively from the archive of the Department of Orthodontics. These images had been required for diagnosis and treatment planning by the orthodontists (impacted, transposed or supernumerary teeth, restricted maxilla, and/or any additional piece of diagnostic information that 2D X-ray photos did not help with). Before obtaining patients’ written informed consent and consent to publish, patients (or next of kin) were informed verbally and in writing about the necessity of taking CBCT images and possible risks.

The sample consisted of 91 CBCT scans of patients (mean age 13.1 ± 2.65 years; range 8.6–17.8 years; 46 females and 45 males). The inclusion criteria were as follows: patients must be aged 8–18 years, no systemic disease or maxillofacial deformities or trauma, and no previous orthodontic treatment.

All selected CBCT scans were performed in the same radiographic clinic using the Scanora 3D device with 85 kV, 15 mA, and 2.25-s exposure time. The field of view was 14 × 7 cm. The scan data were reconstructed with a voxel size of 0.3 mm^3^. Dicoms data were processed with OnDemand® 3D software viewer v1.0 (CyberMed, Finland).

**Fig. 1:**
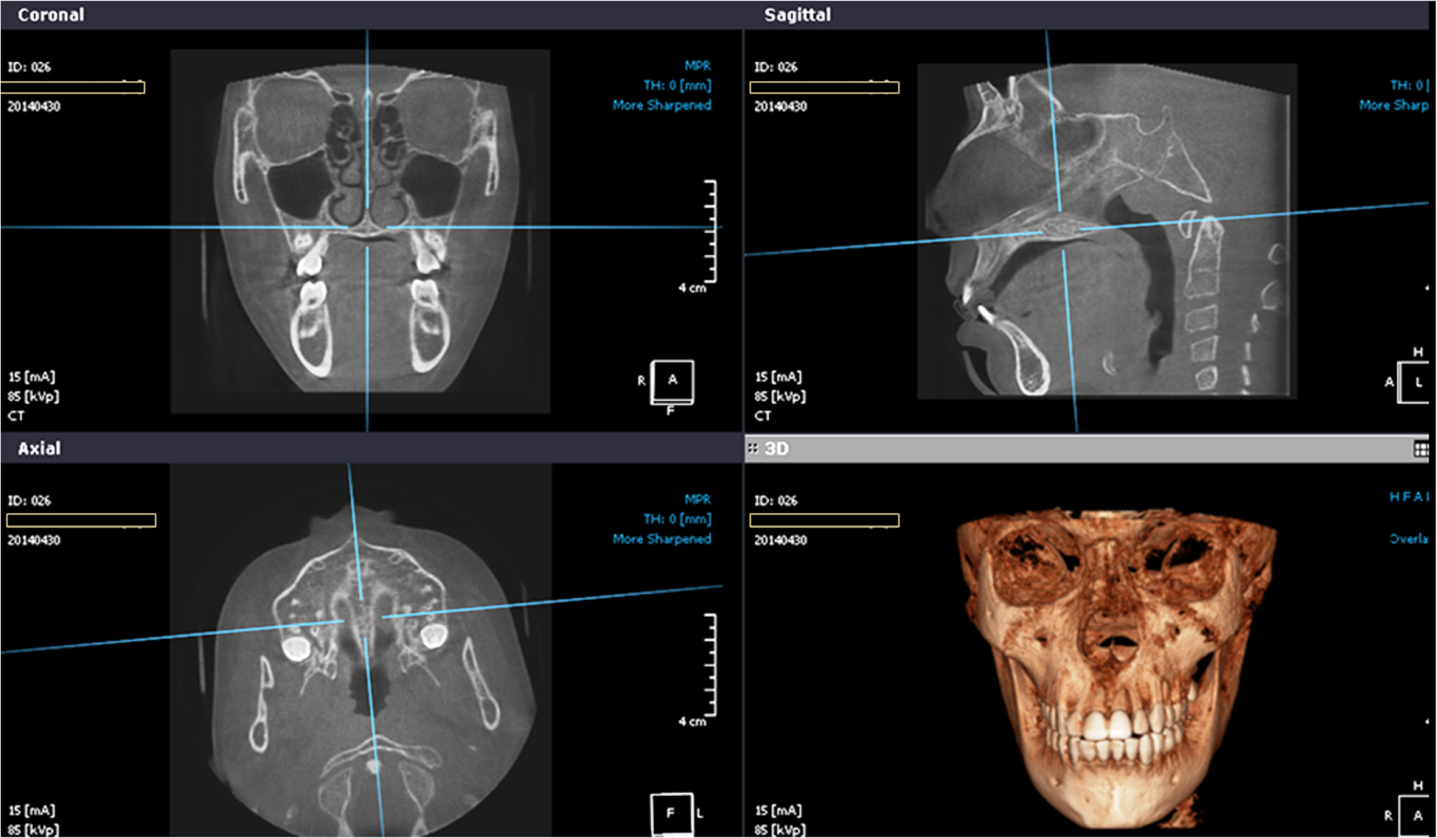
Standardization of CBCT radiograph in axial and sagittal planes

Then, CBCT images were standardized at both the midsagittal slice (the horizontal axis passed through the center of the superoinferior dimension of the hard palate) and the axial slice (the vertical axis passed through anterior and posterior nasal spine) (Fig. 1).

### Classification of morphological maturation stages of MPS

The procedures were performed according to the protocol described by Angelieri et al. [4, 21, 22]. In the sagittal plane, the midsagittal cross-sectional slice was used to position the horizontal axis passed through the center of the superoinferior dimension of the hard palate (from the nasal to the oral surfaces). After that, the most axial central cross-sectional slices were used for sutural assessment (Fig. 1).

**Fig. 2:**
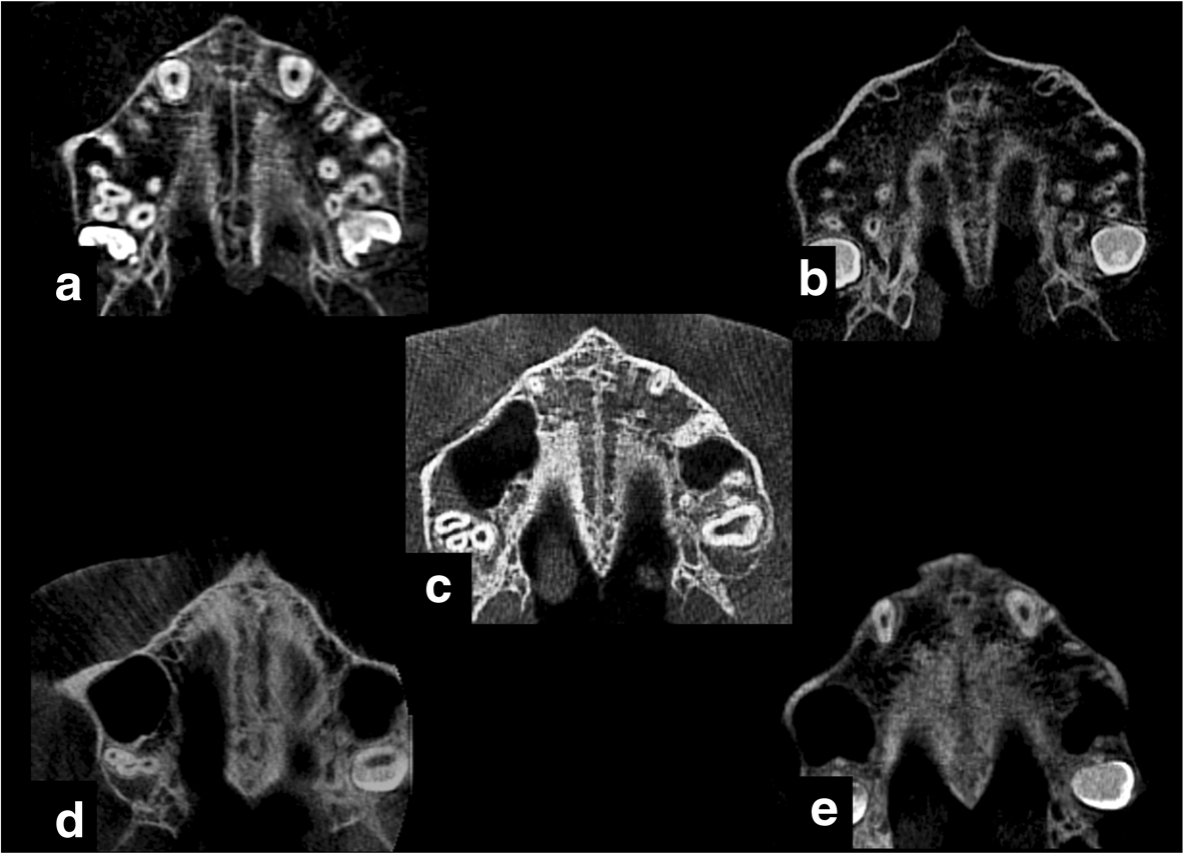
**a**–**e** Morphological maturation stages of midpalatal suture

All slices were randomized in a power point presentation file with a black background and the same monitor conditions. Then, they were conducted blindly by one expert examiner to five stage groups (a, b, c, d, e) according to the method of Angelieri et al. (Fig. 2).

For the subjects who exhibited a thick or curved palate, two axial cross-sectional slices were determined.

To evaluate the intraexaminer agreement, all images were reclassified a month later by the same examiner.

### Bone density measurements

**Fig. 3:**
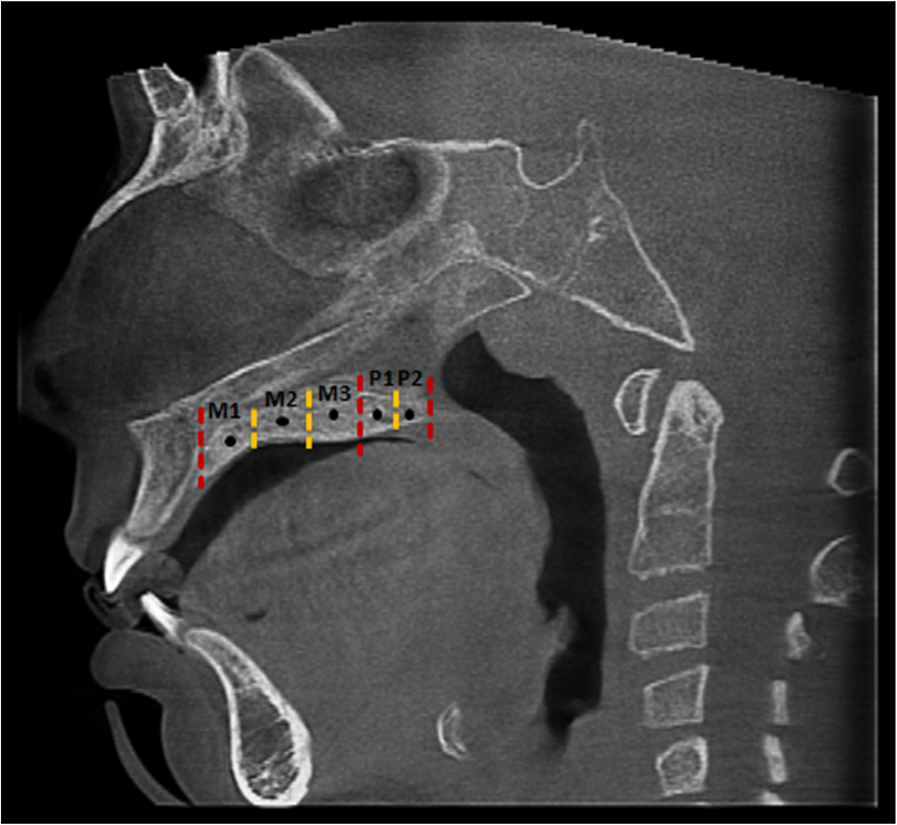
The three equal sections of maxillary region (M1, M2, M3) and the two equal sections of palatal region (P1, P2)

**Fig. 4:**
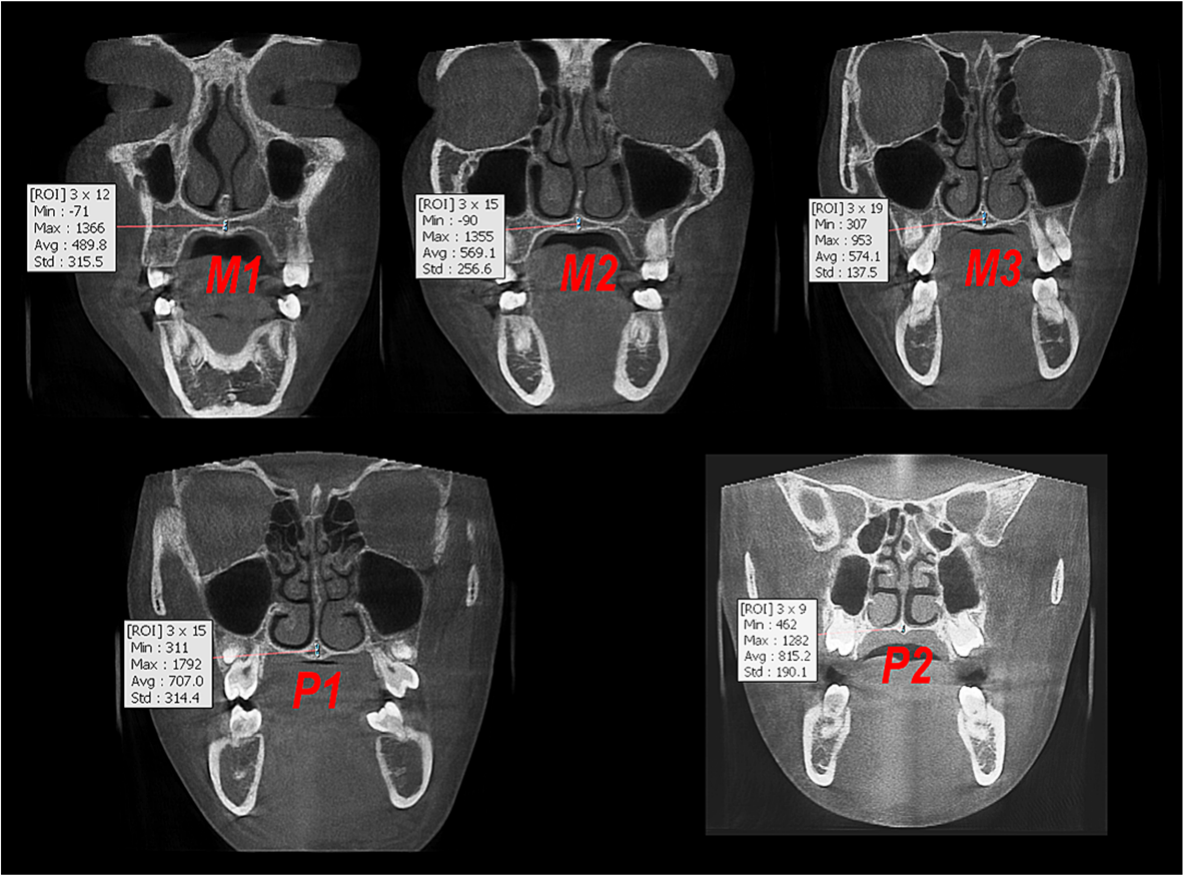
Measurement of midpalatal suture density in the middle of each section

On the midsagittal slice passing through the anterior and posterior nasal spine, the maxillary region was divided into three equal sections (M1, M2, M3), while the palatal region was divided into two equal sections (P1, P2) (Fig. 3). Then, on the coronal slice passing through the middle of each of these sections, the mean MPSD value was measured using a rectangle region of interest (ROI) at 3 mm in width and along the full height of the midpalatal suture (the cancellous bone between the upper and lower cortical bones) (using OnDemand 3D App) (Fig. 4).

Bone density of the midpalatal suture in the maxillary region (MPDm) was the average of the bone density values of its three sections (M1, M2, M3).

Bone density of the midpalatal suture in the palatal region (MPDp) was the average of the bone density values of its two sections (P1, P2).

To test the intraexaminer reliability, 30 images randomly selected were measured a month later by the same examiner.

### Statistical analysis

A weighted kappa coefficient was calculated to evaluate the intraexaminer agreement for the classification of the midpalatal suture maturation stages; agreement was defined in conformity with the scale described by Landis and Koch [27] (< 0, no agreement; 0–0.20, slight agreement; 0.21–0.40, fair agreement; 0.41–0.60, moderate agreement; 0.61–0.80, substantial agreement; 0.81–1.00, almost perfect agreement).

The intraclass correlation coefficient (ICC) was used to assess intraexaminer reliability for MPDS measurements.

Data were analyzed using SPSS 13.0 edition. Kruskal-Wallis and Mann-Whitney *U* tests were used to detect the significant differences in bone density values in the maxillary and palatal regions among the five morphological maturation stages. Statistical significance was determined at *P* < 0.05.

## Results

The weighted kappa coefficient was 0.98, which reveals a high data reproducibility.

The results of the intraclass correlation coefficient (ICC) test revealed a high reliability between the two assessments for all regions (ICC > 0.8).

The sample distribution according to morphological maturation stages was summarized in Table 1.

**Table 1:** Sample distribution according to morphological maturation stages

Morphological maturation stage	Subjects no.	Percentage
A	11	12.1
B	31	34.1
C	19	20.9
D	14	15.4
E	16	17.6
Total	91	100

**Fig. 5:**
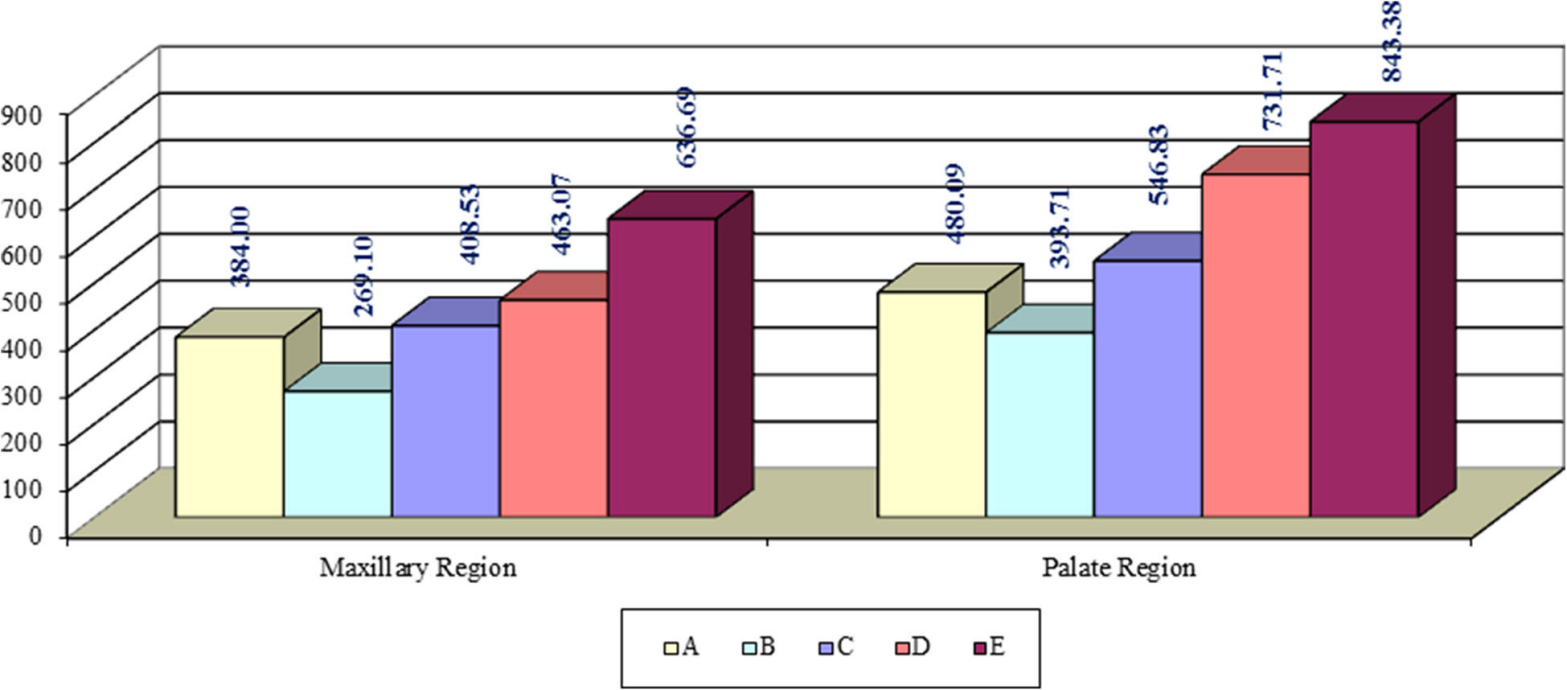
Mean of midpalatal suture density values according to morphological maturation stages

Midpalatal suture densities in maxillary and palatal regions increase with morphological maturation stage advancement (Fig. 5).

Significant differences were found in MPSDm between E stage and A-B-C-D stages and between B stage and C-D stages (Table 2). Significant differences were also found in MPSDp between D-E stages and A-B-C stages and between B stage and C stage (Table 3).

**Table 2:** Differences of midpalatal suture density measurements in the maxillary region among morphological maturation stages

Studied location	Morphological maturation stage		*P* value
Bone density of the maxillary region	A	B	0.116
		C	0.561
		D	0.381
		E	0.003
	B	C	0.035
		D	0.020
		E	< 0.001
	C	D	0.512
		E	0.004
	D	E	0.048

**Table 3:** Differences of midpalatal suture density measurements in the palatal region among morphological maturation stages

Studied location	Morphological maturation stage		*P* value
Bone density of the palatal region	A	B	0.271
		C	0.519
		D	0.002
		E	0.001
	B	C	0.037
		D	< 0.001
		E	< 0.001
	C	D	0.024
		E	0.003
	D	E	0.198

## Discussion

It is difficult for the clinicians to make a decision between RME and SARME to widen the constricted maxilla, especially for adolescents and young adults [3, 18, 26]. Since a great variability is often found in MPS maturation in such patients, chronological age cannot be depended on to predict the outcomes of the treatment. Thus, individual assessment of MPS is essential before the treatment [4, 20]. Investigating whether the morphological maturation stages could reflect the changes of MPSD with maturation may assist to confirm the reliability of this classification to make a clinical decision.

As it is previously stated, according to recent studies, MPSD is one of the most important factors that determine the resistance of MPS to expansion forces [5, 18, 20, 26]. Grünheid et al. [5] confirmed that MPSD is the most important factor to predict the skeletal effects of RME. However, low standardization between CBCT machines exists, and the Hounsfield scale may vary among studies [28]. Therefore, it is difficult to compare the MPDS taken from different CBCT machines or to depend on absolute values to shift from RME to SARME.

In the current study, all CBCT images were taken by one device (Scanora 3D) using the same exposure protocol. A strong linear correlation between the voxel gray values from the Scanora 3D device and the actual Hounsfield units (HU) derived from multislice CT was reported [29]. Moreover, exposure protocols from certain devices show stable gray values that could be related to HU and density [29].

The Angelieri et al. classification of MPS maturation is a simple method and could be a reliable parameter for the clinical decision, and it has a massive advantage that it does not vary between different CBCT machines [4, 22]. But the limitation of this methodology is that direct comparison of the histological morphology to the CBCT morphology of the suture is incompatible and more studies are needed to validate the proposed maturation stages as a gold standard [20].

The descriptive maturation stages of MPS categorized by Angelieri et al. proposes that MPS in the palatal region starts to fuse in D stage and is completely fused in E stage. Similarly, that was proven in the current study, as MPSDp in D and E stages were significantly higher than in A, B, and C stages than those in A, B, and C stages.

MPS in the maxillary region starts to fuse only in E stage. According to the findings of the current study, MPSDm in E stage was significantly higher than those of previous stages (A, B, C, D). Thus, it is supposed that there is a coordination between the developmental changes in the morphology of MPS and the increase of MPDS during maturation. The morphological maturation stages in the mid-thickness of the hard palate as proposed by Angelieri et al. [4, 21, 22] reflect the progress of MPDS in the full thickness of the hard palate.

Angelieri et al. suppose that RME treatment is successful in A and B stages, and it is also successful in stage C with less skeletal effects, but in D and E stages, SARME should be done. The differences in MPDS between these stages, found in the current study, may approve the reliability of Angelieri et al. classification to choose between RME and SARME to make a clinical decision.

Likewise, Kwak et al. found a strong negative correlation between fractal dimension and MPS morphological maturation and confirmed that the reliability of this classification was sufficiently high to justify its use in clinics. They concluded that fractal analysis may be useful for the evaluation of MPS maturation, but this method requires the clinician to have significant familiarity with image processing and possessing necessary software. Moreover, fractal dimensions can differ according to the calculation methods used [20, 30].

From the other point of view, Angelieri et al. classification requires a lot of training to reach an adequate level of ability to distinguish between the five stages. While, on the contrary, MPDS calculation can be achieved easily using CBCT. Unfortunately, the direct comparison of the gray density value between CBCT scanners is not possible [31]; thus, absolute values of MPDS could not be depended on to determine the maturation stage of MPS or to choose between RME and SARME. Future studies to find such values might validate MPDS as an objective, quantitative, simpler, and overall more useful indicator. However, since the morphological maturation stages of the midpalatal suture could reflect the changes of its bone densities during maturity as shown by the current study, this might support the suggestion of using Angelieri et al. classification to choose between RME and SARME in the critical cases of adolescents and young adults.

Although MPS is the most important definitive factor to determine the success of RME [11], one must keep in mind the other important resistance regions. However, more research is needed to determine the effect of the development of these regions on maxillary resistance to RME.

## Conclusions

The differences in MPDS among the morphological maturation stages support their reliability in clinical application to choose between RME and SARME.

Since the direct comparison of the gray scale value between CBCT scanners is not possible, morphological maturation stage method seems to be more practical and reliable than bone density one.

## Abbreviations

CBCT: Cone beam computed tomography
MPS: Midpalatal suture
MPSD: Midpalatal suture density
MPSDm: Midpalatal suture density in maxillary region
MPSDp: Midpalatal suture density in palatal region
MPSM: Morphological maturation of midpalatal suture
OI: Obliteration index
RME: Rapid maxillary expansion
SARME: Surgically assisted rapid maxillary expansion
